# Model Comparison for Breast Cancer Prognosis Based on Clinical Data

**DOI:** 10.1371/journal.pone.0146413

**Published:** 2016-01-15

**Authors:** Sabri Boughorbel, Rashid Al-Ali, Naser Elkum

**Affiliations:** 1 Biomedical Informatics Division, Sidra Medical and Research Center, Doha, Qatar; 2 Clinical Epidemiology Division, Sidra Medical and Research Center, Doha, Qatar; Qom University, ISLAMIC REPUBLIC OF IRAN

## Abstract

We compared the performance of several prediction techniques for breast cancer prognosis, based on AU-ROC performance (Area Under ROC) for different prognosis periods. The analyzed dataset contained 1,981 patients and from an initial 25 variables, the 11 most common clinical predictors were retained. We compared eight models from a wide spectrum of predictive models, namely; Generalized Linear Model (GLM), GLM-Net, Partial Least Square (PLS), Support Vector Machines (SVM), Random Forests (RF), Neural Networks, k-Nearest Neighbors (k-NN) and Boosted Trees. In order to compare these models, paired t-test was applied on the model performance differences obtained from data resampling. Random Forests, Boosted Trees, Partial Least Square and GLMNet have superior overall performance, however they are only slightly higher than the other models. The comparative analysis also allowed us to define a relative variable importance as the average of variable importance from the different models. Two sets of variables are identified from this analysis. The first includes number of positive lymph nodes, tumor size, cancer grade and estrogen receptor, all has an important influence on model predictability. The second set incudes variables related to histological parameters and treatment types. The short term vs long term contribution of the clinical variables are also analyzed from the comparative models. From the various cancer treatment plans, the combination of Chemo/Radio therapy leads to the largest impact on cancer prognosis.

## Introduction

Cancer is the leading cause of death world-wide, accounting for 13% of all deaths [1]. For women, breast cancer is one of the major causes of death, in both developed and developing countries [2]. In 2012, the number of breast cancer cases worldwide was estimated at 14.1 million new cases and 8.2 million deaths. It is estimated that incidence of breast cancer has increased by 20% since 2008, and mortality by 14% [3]. Disease management of breast cancer is a complex process and the treatment plan depends largely on cancer prognosis. Therefore the estimation of the prognosis period is an important information for both patients and clinicians. Cancer prognosis can be defined as the estimation of the probability of surviving beyond a certain period of time. For example, a 5-year prognosis of 80% would mean that the chance of surviving 5 years after cancer diagnosis, or surgery, is estimated as a 80% probability. The prediction of patient prognosis can be very useful for the selection of best treatment protocols. Eloranta et al. introduced a relative survival framework to estimate the probability of death in the presence of competing risks [4]. In this work we formulate the prognosis estimation problem in terms of a classification problem. For different prognosis periods (e.g., 5 or 10 years), classification classes are defined using patient survival information. Patients who survived beyond the prognosis period are labeled in the positive class and patients who died before reaching that period are considered in the negative class. Hence, a binary classification problem can be properly defined and predictive models from machine learning can be used. We made the choice to focus this research on predictive model comparisons and we excluded survival analysis models (such as Cox proportional hazard models) from the scope of this research. The no-free lunch theorem states that without prior knowledge about the prediction problem there is no single model that will always perform better than others [5]. Therefore, we opt for the approach of considering multiple predictive models for the prognosis of breast cancer. In the literature there are a number of references that investigated the comparison of multiple machine learning techniques for the prediction of breast cancer prognosis. Maglogiannis et al. propose five feature models based on clinical, gene expression and combined models are evaluated under different conditions [6]. Binary classifiers (SVM, Random Forests and Logistic regression) are tested on the five models for the prognosis task. A comparison of three prediction algorithms (Decision trees, Artificial Neural Networks and logistic regression) are given in [7]. Data with 200,000 samples from SEER are used for the evaluation. The three methods performed with 93.6%, 91.2% and 89.2% accuracy, respectively. Burke et al. evaluated different predictive models including pTNM staging, PCA, CART decision tree (shrunk, pruned), ANN (probabilistic, back-propagation, etc) on 8,271 samples for 5-year prognosis end-point [8]. The performance in terms of area under curve of the receiver operating characteristic AU-ROC ranged from 0.71 to 0.78. The best reported model is the ANN-back-propagation. A comparison of seven algorithms for the same task on 37,256 subjects showed that decision tree J48 had the highest sensitivity, and Artificial Neural Network had the highest specificity [9].

Here we evaluate and compare the most recent and successful predictive techniques in machine learning. We consider the area under the ROC curve (AU-ROC) as the performance metric for the analysis. Maximizing AU-ROC allows us to avoid the problem of choosing a single operating point for the classification model. The latter requires an additional validation dataset, or should be properly integrated in the training and validation stage. In addition, choosing a classifier threshold would limit the utility of the method. For instance, if the predictive methods are planned to be used in a cancer screening system, specificity should be very high, even at the cost of sensitivity. While in a diagnostic system, both sensitivity and specificity should be high. Since we would like to capture many potential clinical applications, maximizing AUC-ROC allows us to keep all these applications feasible.

Below we provide a description of the dataset and pre-processing steps applied in order to clean and format the data. The chosen models are then presented and a description of model fitting and parameter tuning results are given. Finally, model comparison is analyzed using statistical tests, and contribution of the clinical variables to the model prediction is discussed.

## Data pre-processing

The analysis we describe in this paper is based on the clinical subset of the METABRIC breast cancer dataset [10]. Our aim was to restrict the analysis to the most common variables in clinical practice. For this reason we excluded genomic related variables as these are not yet widely available for clinicians and might limit the applicability of our approach. Table 1 provides the list of included variables as well as description details and possible values.

**Table 1:** Description of the clinical variables included in the comparative analysis.

Variables	Description	Value examples
Age at diagnosis	Age at cancer diagnosis	42.7 years
Tumor size	Tumor size in mm (used in TNM classification)	23, 41 cm
Lymph nodes positive	The number of lymph nodes (used in TNM classification)	1, 3, 10
Grade	Grouping of Nottingham scores into three groups	1, 2, 3
Histological type	Histology outcome	DCIS, IDC, ILC
Estrogen Receptor IHC status	Estrogen receptor status measured by Immunohistochemistry	positive, negative
PR Expr	Progesterone Receptor Expression	positive, negative
Her2 Expr	Human Epidermal growth factor receptor 2 (Her2)	positive, negative
Treatment	One of the three treatments (CT: Chemotheraphy, HT: Hormonal Therapy, RT: Radiaton Therapy)	CT, CT/, CT/HT/RT, NONE, RT
Stage	TNM stage	1, 2, 3, 4
Lymph nodes removed	Number of removed lymph nodes	8, 14, 25

The first step in building a predictive model is a pre-processing stage. It consists typically in cleaning and formatting the data before model fitting. For missing values we decided to remove the related subjects. Although imputation techniques could have been used for dealing with the missing values, we opted for not introducing an additional bias factor to the analysis due to the influence of the imputation technique choice on the prediction results. In addition, the number of subjects after missing values removal was still sufficiently large (*N* = 1,421) for a good statistical analysis.

The next step in the pre-processing stage is to transform categorical variables into binary dummy variables. This is required for a number of predictive models as they only use numerical or binary input variables. Variable collinearity is also checked to ensure no strong correlation between the predictors. Since some models work under the assumption that variables are independent, highly correlated variables would invalid the results of these models. Collinear variables are obtained by decomposing the data matrix using a QR decomposition to verify if the matrix is full rank then find the set of variables that are collinear by iteratively removing one variable and checking the matrix rank. We excluded three histological variables due to their collinearity namely: “MIXED NST AND A SPECIAL TYPE”, “OTHER INVASIVE” and “IDC-MUC”. The last step was to center and scale the data as most models are affected by the difference in scaling of the variables. The normalization step is not applied at a global level of the data, but rather at each resampling data block to avoid over-fitting.

Next, we defined different subsets of the data to solve the prognosis prediction problem. Given a prognosis period *T*, subjects that survived longer than *T* are labeled in the class {*survived*} and subjects that did not survive longer than *T* are labeled in the class {*not survived*}. Subjects that are indicated to have survived with a period *t* < *T* are discarded from the subset. The choice of *T* depends on the short term vs. long term prediction to be achieved by the models. For this analysis we tried to cover both short and long terms prediction by choosing four values of the prognosis period *T*, namely 2, 5, 8 and 11 years. For each data subset, the prognosis prediction can be formulated in term of a binary classification problem. Table 2 summarizes the sample distribution among the class for the different periods *T*, as well as the total sample size. We notice that the total number of samples decreases as the prognosis period *T* increases. If we consider the 2- and 5-year prognosis periods as examples, there is a decrease of 158 subjects in the total number of samples between these periods. These subjects are reported to have survived between 2 and 5 years at the end of the clinical trial. Therefore they cannot be placed in any of the two classes for the 5-year period and hence are excluded from the analysis for that period. This is referred to as random censoring in survival analysis [11].

**Table 2:** Sample size for the different selected prognosis periods. Depending on the surival period, samples are included or excluded in one or more datasets among the four.

Prognosis period *T*	#Survived > T	#Not survived > T	Total #samples
2 years	1311	76	1387
5 years	976	253	1229
8 years	674	343	1017
11 years	434	398	832

## Model comparison

We investigate the comparison of classification models for the purpose of predicting breast cancer prognosis. Our selection of models was based on diversifying the choice to capture a wide spectrum of methodologies and levels of model complexity (linear models, decision tree, neural networks, kernel methods). We selected techniques from linear modeling; Partial Least Square (PLS), Generalized Linear Model (GLM), and a penalized version of GLM namely Elastic-Net. We included a Neural Network model as it is an important class of non-linear predictive models. From kernel methods, we selected Support Vector Machine with a Gaussian kernel as it is capable of dealing with non-linearity and data noise. From decision tree methods, Random Forests and Boosted trees are considered in this analysis since they are well-known ensemble-based decision-tree techniques. Finally, we included a basic prediction technique, i.e. k-Nearest Neighbors, to verify the complexity of the prediction problem.

### Models description

We describe the technical aspects of the selected predictive models and give the main optimization and prediction equations. This gives a general idea on how these models are fitted and identifies the tuning parameters that need to be estimated for an optimal use of the models. The list of tuning parameters and notations are summarized in Table 1. For further details on the models we refer the readers to a key publication for each model.

- **Neural Networks**: NNs introduce intermediate variables that are not observable, called hidden variables or hidden units *h*_*k*_ [12]. These variables are obtained by combining the input variables linearly and transforming them using a sigmoid function as $h_{k}\left(x\right)=g(\beta_{0k}+\sum_{i=1}^{P}x_{i}\beta_{jk})$ where $g\left(v\right)=\frac{1}{1+e^{-v}}$ and *P* is the number of input variables. The number of hidden variables *H* is usually a tuning parameter. The prediction function can be written as a linear combination of the hidden variables $f\left(x\right)=\gamma_{0}+\sum_{k=1}^{H}\gamma_{k}h_{k}\left(x\right)$. Fitting the function *f* can be achieved in a robust way by introducing a regularization term with a weight decay parameter *λ*. The problem then consists of finding the coefficients *β*_*jk*_ and *γ*_*k*_ that optimize the squared errors: $\sum_{i=1}^{n}{\left(y_{i}-f\left(x_{i}\right)\right)}^{2}+\lambda \sum_{k=1}^{H}\sum_{j=0}^{P}\beta_{jk}^{2}+\lambda \sum_{k=0}^{H}\gamma_{k}^{2}$ where *n* is the sample size and (**x**_*i*_, *y*_*i*_) is the training dataset.

The higher the value of the weight decay *λ*, the lower the risk of over-fitting, since the obtained solution is smoother. A back-propagation algorithm can be used to optimize this problem where *λ* is a tuning parameter. However there is no guarantee that this optimization will lead to a global solution, as convexity is not assured. The model above is called a single-layer feed-forward neural network. More advanced neural networks have been proposed where multiple hidden layers and customized connections between the units are introduced.

- **Support Vector Machine**: SVM can be formulated in the context of binary classification as the model that finds the decision boundary which maximizes the margin between two data classes [13]. The dual optimization problem can be written as a convex problem: $\tilde{L}\left(\alpha \right)=\sum_{i=1}^{n}\alpha_{i}-\frac{1}{2}\sum_{i,j}\alpha_{i}\alpha_{j}y_{i}y_{j}k\left(x_{i},x_{j}\right),\;\text{subject to}\;0\leq \alpha_{i}\leq C\;\text{and}\;\sum_{i=1}^{n}\alpha_{i}y_{i}=0$. The convexity of the problem ensures a unique global solution. The second constraint ensures the sparsity of the solution. Hence only a few training samples will be expressed in the prediction function: *f*(**x**) = ∑_*i* ∈ *SV*_ *α*_*i*_ *k*(*x*_*i*_, **x**) where *SV* is the set of support vectors. These properties ensure an efficient prediction function for SVM. The introduction of kernel functions allows decoupling of the prediction and generalization properties from data modeling and representation. For our analysis we chose to use Gaussian kernel $K_{\sigma }\left(x,y\right)=e^{\frac{\left||x-y|\right|}{\sigma }}$. The Gaussian kernel can deal with non-linearity in the data, in addition to scaling. The tuning parameters *σ* and *C* are optimized via resampling procedures.
- **Random Forests**: Decision-tree techniques seek, in an iterative manner, the best variables and disjoint regions that lead to a minimum training error when the decision is taken as the average outcome on these regions. The basic optimization problem of decision trees can be written as follow $\sum_{x_{i}\in R_{1}}{\left(y_{i}-{\hat{y}}_{R_{1}}\right)}^{2}+\sum_{x_{i}\in R_{2}}{\left(y_{i}-{\hat{y}}_{R_{2}}\right)}^{2}$. The process of looking for regions and selecting variables is performed in a nested way until some stopping criterion is fulfilled such as a minimum number of points in each region. Techniques for pruning the fully grown trees are applied to create robust decision trees. Ensemble techniques have been applied to solve the sensitivity of decision trees to different views of data. Random Forests present an improvement on simple decision tree by applying an ensemble technique in a more optimised way than the bagged trees model [14]. The latter uses bootstrapping to average the tree decisions. However, the obtained trees are highly correlated. Random Forests solve this issue by randomly picking a limited number of variables for each bootstrap iteration. The number of randomly selected variables *mtry* is typically a tuning parameter for Random Forests.
- **Boosted Trees**: In Random Forests and bagging tree, the different trees are combined in an independent fashion. Boosted trees, however, update the decision trees at each iteration [15]. The tuning parameters for boosted trees are the interaction depth, which defines the tree depth, and the number of iterations. In general, boosting has been shown to work well with weak-learners, therefore it is expected that shallow trees (low interaction depth) should give better performances than deep trees.
- **GLM-Elastic Net**: Generalized Linear Models (GLM) are an extension of regression models designed to deal with error distributions beyond the normal distribution. GLM-Elastic Net is a penalized version of GLM [16]. The fitting of GLM-Net model fitting consists in optimizing the binomial likelihood function $\ell \left(\theta \right)=\sum_{i=1}^{N}log\left(\right)-\lambda [\left(1-\alpha \right)\frac{1}{2}\sum_{j=1}^{P}\beta_{j}^{2}+\alpha \sum_{j=1}^{P}\left|\beta_{j}\right|]$ where *α* is a mixing factor that determines the weight between the lasso regression (*α* = 1) and the ridge regression (*α* = 0), and *λ* is a global regularization parameter. The basic GLM included in this analysis can be seen as a particular case of GLM-Elastic Net with *λ* = 0. We made the choice of including the simple GLM to evaluate the effect of introducing the regularization term on performance in the case of breast cancer prognosis prediction.
- **k-Nearest Neighbors**: Nearest-Neighbor algorithm is one of the simplest classification algorithms as its implementation is straightforward. k-NN uses the training samples to predict a new sample by a majority vote on the outcome of the k-nearest points to the new sample. This decision function can be written as $\hat{y}\left(x\right)=\frac{1}{k}\sum_{x_{i}\in N_{k}\left(x\right)}y_{i}$, where *N*_*k*_(**x**) is the training data subset of k nearest points to **x**. Neighborhood searching implies the use of a metric such as Euclidean distance. The number of neighbors *k* is a tuning parameter. The higher *k*, the smoother the decision boundaries. We chose to include k-NN in this analysis to identify how the performance of advanced predictive models compare with a basic one. This helps assessing the level of difficulty of the classification problems in hand.
- **Partial Least Square**: Principal Component Analysis (PCA) is similar in concept to Partial Least Squares (PLS) [17]. PCA seeks linear combinations of the input variables that maximize their variation. These linear combinations are called components. A few first components usually capture most of the variation in the variables. PCA is an unsupervised dimension reduction technique, and hence cannot be used for classification. PLS extends the PCA approach for supervised learning. It seeks optimal components that both maximize variable variation and correlation with the outcome variable. We note that there is a difference between LDA and PLS. In LDA there are two steps, applying PCA then maximizing the between-to-within group variability. In PLS, the two steps are combined in one objective function.

### Model parameter tuning

Data resampling techniques are used for model evaluation. Approaches such as cross-validation, leave-one-out or bootstrapping are typically used for this purpose. Resampling techniques are used for the tuning of model parameters [18, 19]. These approaches ensure that the performance estimate is not overly optimistic. First, a grid of possible values of the tuning parameters are defined. Then the data is split into training and hold-out sets, therefore defining multiple resampling iterations. For each tuning parameter value, the model is fitted using the training data and prediction is performed on the hold-out set. The average performance can be calculated across the different resampling iterations. The previous step is repeated across the tuning parameter set. The optimal tuning parameter is selected as the one that corresponds to the best performance.

In our analysis we modify the previously described approach to take into account both parameter tuning and final performance estimation. As described in algorithm 1, we split the data randomly into four folds such the two class proportions are kept the same across the splitting. This step was repeated randomly 10 times to define 10 resampling iterations. We used the same resampling to evaluate the different models. Hence we ensure a fair model comparison by having the same data splits and repetitions. Within each repetition, the four data folds are used as follows; 1) the first two folds are used to select the optimal tuning parameters, 2) the second two folds are used to evaluate the performance of the model based on the selected tuning parameters.

**Algorithm 1**: Model training, parameter tuning and performance estimation

1 Define sets of model parameter values to evaluate

2 Prepare data resampling: split data using stratification into 4 folds with 10 repetitions

3 **for** *each parameter set* **do**

4  **for** *each resampling iteration* **do**

5   Fit the model on fold1 data

6   Predit fold2 data samples

7  Calculate the average performance across repetitions from fold2 sample predictions

8 Determine the optimal parameter set

9 Fit the model on fold3 samples

10 Predict fold4 data samples

**Fig 1:**
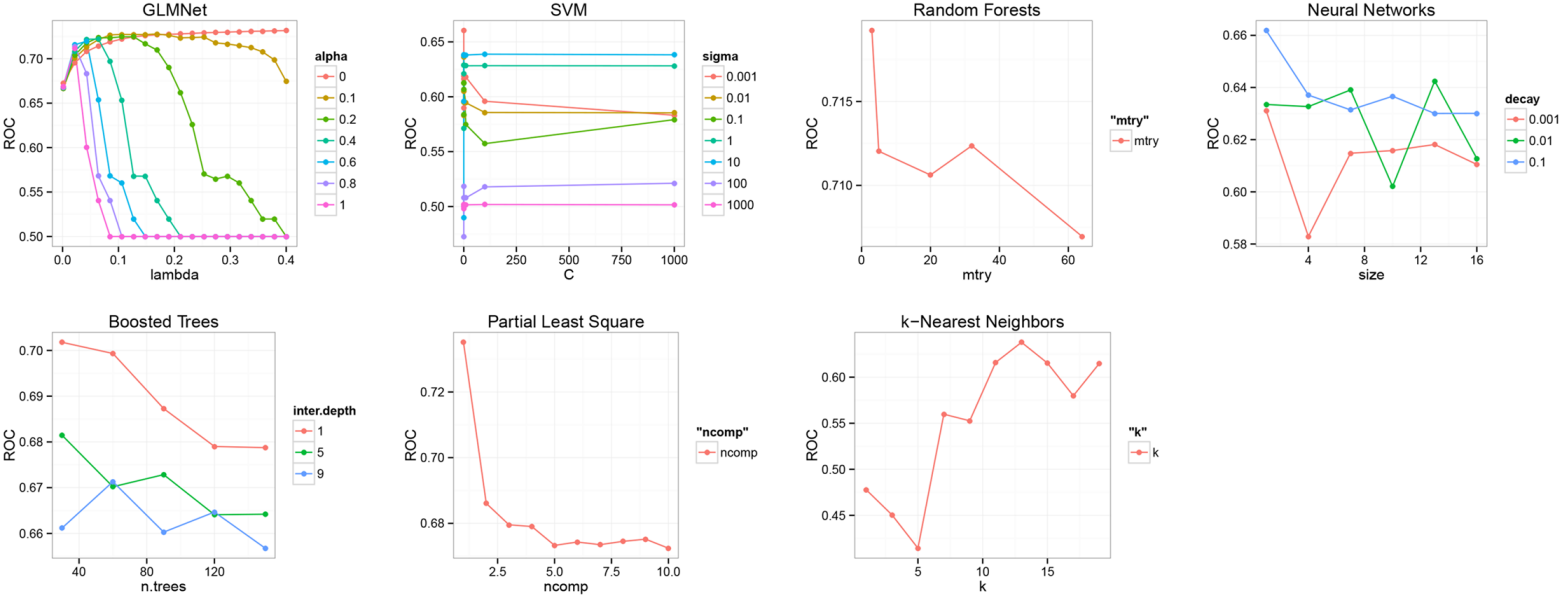
Tuning results of model parameters using re-sampling (GLM is not included since it does not have tuning parameters).

Table 3 summarizes the tuning parameters of the different models. There is one model without tuning parameters (GLM), three models with one tuning parameter (PLS, RF and k-NN) and four models with two tuning parameters (NN, B-Trees, GLMNet, SVM). We mention that shrinkage parameter for boosted trees has been considered fixed in order to control model complexity. For each set of parameters, the models are fitted and evaluated using the procedure described in algorithm 1. This procedure is repeated for the complete set of hyper-parameters. The optimal parameters for each model is selected by maximizing the performance based on a grid of parameter values. Fig 1 shows the average AU-ROC for the different models and parameters. SVM seems to be insensitive to the cost choice *C*, kernel smoothing parameter *σ* of 10 is the optimal. For PLS, the choice of 5 components is optimal. For k-NN, a relatively large number of *k* as 13 is the optimal. For RF, a limited number of randomly selected predictors of 3 seems to give the best performance. As expected for boosted trees, shallow trees (with interaction depth of 1) and a number of iterations (30) were the best parameters.

**Table 3:** Tuning parameters of the listed predictive models.

Models	Tuning parameters
Partial Least Square (PLS)	*ncomp*(#Components)
Neural Networks (NN)	*size*(#hidden units), *decay* (weight decay)
Support Vector Machine (SVM)	*σ*(gaussian kernel), *C* (Cost)
Boosted Trees (B-Trees)	*shrinkage*, *max*.*treedepth*, *boosting*.*iterations*
Random Forests (RF)	*mtry*(#randomly selected variables)
GLMNet	*α*(mixing percentage), *λ*(regularization parameter)
GLM	no tuning parameter
k-Nearest Neighbors (k-NN)	*k*(#Neighbors)

## Results and Discussion

### Model comparison

**Fig 2:**
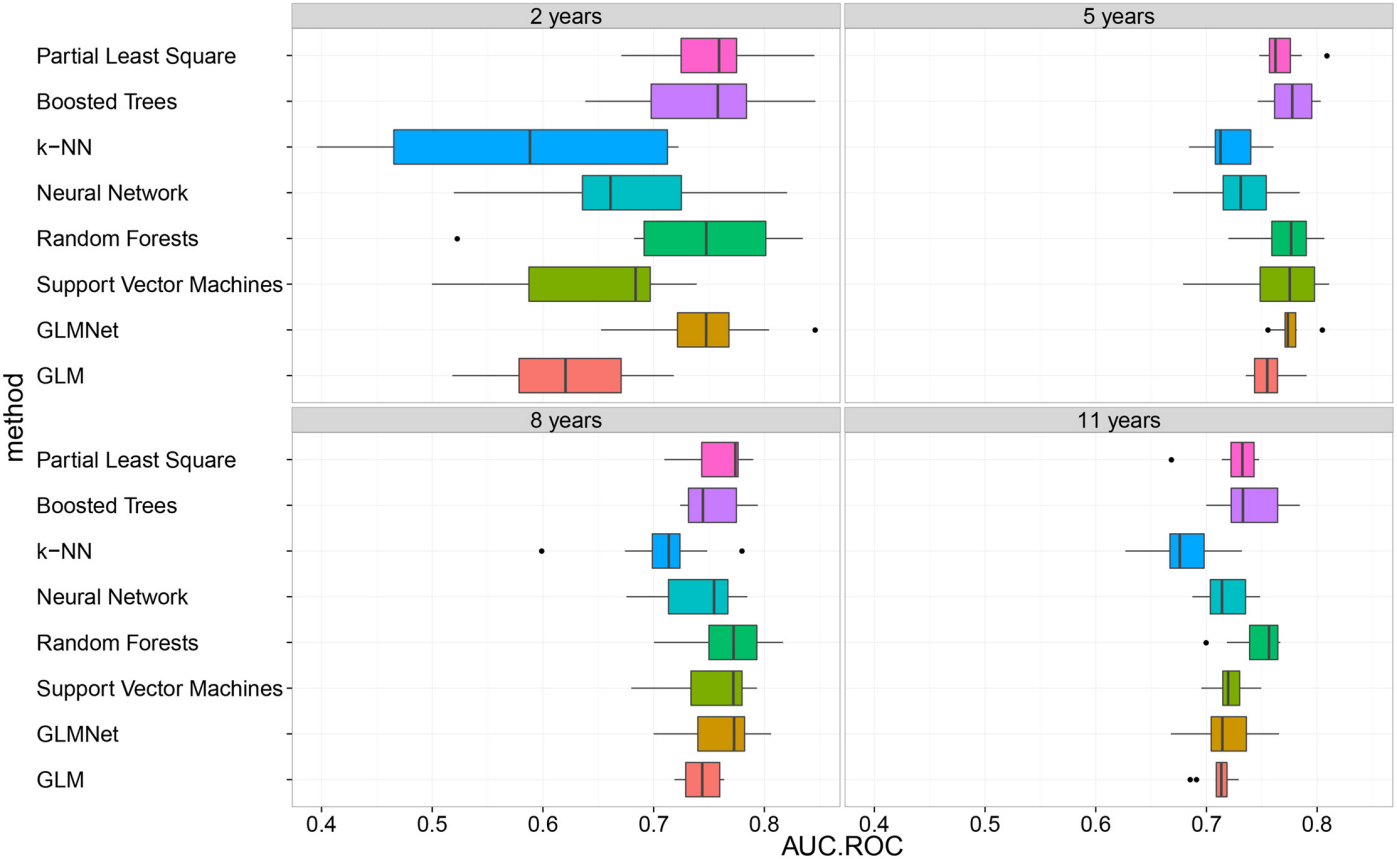
Comparison of model performances for the different use cases (prognosis periods of 2, 5, 8 and 11 years).

Fig 2 depicts boxplots of the 8 model performances in terms of AU-ROC for the different prognosis periods. The choice of AU-ROC as performance indicator is justified for two reasons. Firstly, the data classes can be imbalanced as indicated in Table 2 for the prognosis period of 2 years. Therefore performance criteria such as accuracy is not adequate for assessing model performance as it tends to give advantage to models that always output the class with highest frequency. Secondly, AU-ROC is independent of cut-off point choices and hence keeps the choice of clinical applications open beyond this analysis. k-NN had the worst performance in the four different use cases. This result is expected as k-NN is very sensitive to data sampling and the number of neighbors. Including k-NN in our analysis provided an indication on the lower bound of performance and hence the level of difficulty of the prediction problem in hand. The lowest performance, as depicted in Table 4, is around 58% AUC for 2-year prognosis window. The lower bound is higher for 5 and 8 years and is about 72% AUC. This indicates that it is better to use these prediction techniques for a mid-term prognosis, rather than short or long-term prognosis. This observation is analyzed in more detail below.

**Table 4:** Performance summary in terms of AUC-ROC.

Model	2 years	5 years	8 years	11 years
GLM	0.62 ± 0.07	0.76 ± 0.02	0.74 ± 0.02	0.71 ± 0.01
GLMNet	0.75 ± 0.05	0.77 ± 0.01	0.76 ± 0.03	0.72 ± 0.03
Support Vector Machines	0.64 ± 0.08	0.77 ± 0.04	0.76 ± 0.04	0.72 ± 0.01
Random Forests	0.73 ± 0.09	0.77 ± 0.02	0.77 ± 0.03	0.75 ± 0.02
Neural Network	0.67 ± 0.09	0.73 ± 0.03	0.74 ± 0.04	0.72 ± 0.02
k-NN	0.58 ± 0.13	0.72 ± 0.02	0.71 ± 0.05	0.68 ± 0.03
Boosted Trees	0.75 ± 0.07	0.78 ± 0.02	0.75 ± 0.03	0.74 ± 0.03
Partial Least Square	0.75 ± 0.05	0.77 ± 0.02	0.76 ± 0.03	0.73 ± 0.02

For the prognosis period of 2 years, we notice a large performance variance between the models, as well as large model variances across resampling iterations. This could be explained by the data imbalance between the two data classes (5%, 95%) as described in Table 2. Model variance is lower for the other use cases due to the balance between the two data classes as well as the large number of samples. Table 4 shows a numerical representation of the performance summary as in Fig 2. Mean AUC and standard deviation are captured across the different resampling sets. For 2-year window, GLMNet, Boosted Trees and Partial Least Squares have the best performance (0.75 AUC). For 5 years, Boost Trees model has the highest performance with 0.78 AUC. For 8 and 11 years, Random Forests has the highest performances with 0.77 and 0.75 AUC respectively. In order to assess the stability of model prediction across the different prognosis periods we average model performance as shown in Table 5. Random Forests, Boosted Trees, Partial Least Square and GLMNet have the best performance around 0.75 AUC. In order the assess model predictability over time, we averaged the performance of the top four mentioned models as depicted in Table 6. We observe that the prediction for 5 and 8 years are better than 2 and 11 years with 3% to 4% AUC on average. We can conclude that mid-term prediction (5 and 8 years) is more accurate than short and long-term prediction (2 and 11 years). 5-year prognosis prediction has the highest performance and therefore a 5-year prognosis is the recommended time window from our analysis.

**Table 5:** Overall model performance average in terms of AUC-ROC.

Model	AUC
Random Forests	0.76 ± 0.05
Boosted Trees	0.75 ± 0.04
Partial Least Square	0.75 ± 0.04
GLMNet	0.75 ± 0.04
Support Vector Machines	0.72 ± 0.07
Neural Network	0.71 ± 0.06
GLM	0.71 ± 0.06
k-NN	0.67 ± 0.09

**Table 6:** Performance average of top models sorted by AUC-ROC for the different prognosis periods.

Period	AUC
5 years	0.77 ± 0.02
8 years	0.76 ± 0.03
2 years	0.74 ± 0.07
11 years	0.73 ± 0.03

**Fig 3:**
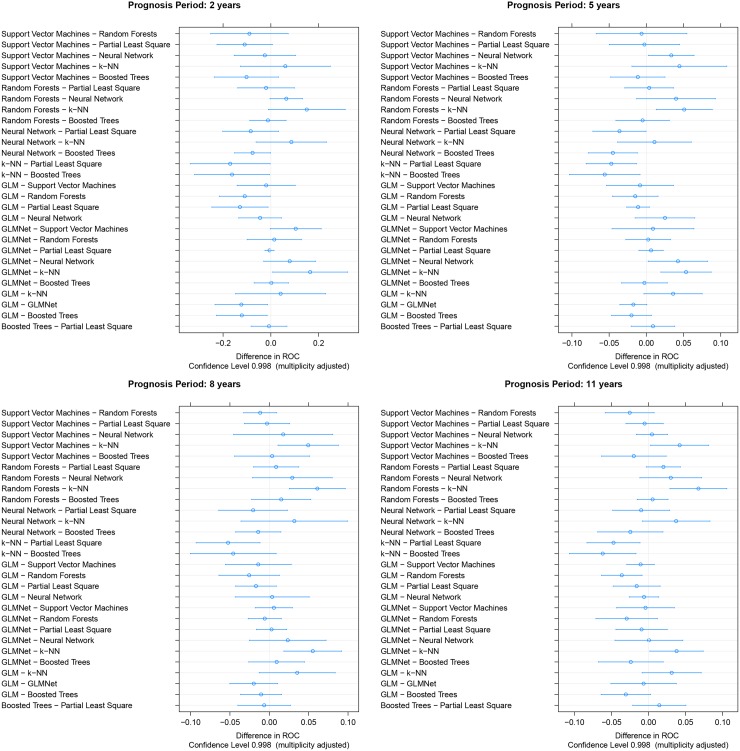
Model comparison in terms of AU-ROC differences and confidence intervals for the different prognosis periods.

In order to better assess model comparison we used a statistical test on model performance differences as proposed in [20, 21]. Paired t-test is applied for each of the model differences across resampling iterations. Fig 3 depicts the average AUC differences of all model pairs and corresponding confidence intervals for the four prognosis periods. We notice that k-NN results are mostly on the left side indicating poor performance compared with the other models. Models such as Random Forests, GLMNet, Boosted Trees and Partial Least Square (PLS) are mostly on the right side indicating superior performance than other models.

Table 7 presents details on AU-ROC average differences and p-value of the paired t-test. The table should be read as follow: For each pair models (row, column), the average of the difference model_row—model_column is given in the upper part of the table. The lower part gives the corresponding p-value for the paired t-test. We highlighted in the upper part performance differences corresponding to p-values below 0.05. As an example, we consider the column corresponding to Boosted Trees in Table 7. We notice that most of the differences are negative, meaning that almost always Boosted Trees have better performances than the other models. Boost Trees model has advance performance varying between 0-8% AU-ROC, with respect to the other models. However this difference is mostly not statistically significant. On the other hand, most of the statistical significant differences involve k-NN as its performance is clearly lower than the rest of the models.

**Table 7:** Paired t-test for the statistical evaluation of model differences for the different prognosis periods. The upper part of the matrix represents the average difference in term of AU-ROC between models in the rows and models in the columns. The lower part represents the adjusted p-values.

2 years	GLM	GLM-Net	SVM	RF	NN	k-NN	B-Trees	PLS
GLM	–	-0.12	-0.02	-0.11	-0.04	0.04	-0.12	-0.13
GLM-Net	0.02	–	0.10	0.01	0.08	**0.16**	0.00	-0.01
SVM	1.00	0.06		-0.09	-0.03	0.06	-0.10	-0.11
RF	0.04	1.00	1.00	–	0.07	0.15	-0.01	-0.02
NN	1.00	0.32	1.00	0.07	–	0.09	**-0.08**	-0.09
k-NN	1.00	0.04	1.00	0.08	0.89	–	-0.16	**-0.17**
B-Trees	0.02	1.00	0.25	1.00	0.05	0.04	–	-0.01
PLS	0.03	1.00	0.07	1.00	0.32	0.04	1.00	–
5 years	GLM	GLM-Net	SVM	RF	NN	k-NN	B-Trees	PLS
GLM	–	-0.02	-0.01	-0.01	0.03	0.04	-0.02	-0.01
GLM-Net	0.06	–	0.01	0.00	0.04	**0.05**	-0.00	0.01
SVM	1.00	1.00	–	-0.01	**0.03**	0.04	-0.01	-0.00
RF	1.00	1.00	1.00	–	0.04	**0.05**	-0.01	0.00
NN	0.65	0.03	0.03	0.26	–	0.01	-0.04	**-0.04**
k-NN	0.08	0.00	0.39	0.01	1.00	–	**-0.06**	**-0.05**
B-Trees	0.27	1.00	1.00	1.00	0.01	0.02	–	0.01
PLS	0.32	1.00	1.00	1.00	0.05	0.01	1.00	–
8 years	GLM	GLM-Net	SVM	RF	NN	k-NN	B-Trees	PLS
GLM	–	-0.02	-0.01	-0.03	0.00	0.04	-0.01	-0.02
GLM-Net	0.53	–	0.01	-0.01	0.02	**0.06**	0.01	0.00
SVM	1.00	1.00	–	-0.01	0.02	**0.05**	0.00	-0.00
RF	0.48	1.00	1.00	–	0.03	**0.06**	0.01	0.01
NN	1.00	1.00	1.00	0.92	–	0.03	-0.01	-0.02
k-NN	0.30	0.00	0.01	0.00	1.00	–	-0.05	**-0.05**
B-Trees	1.00	1.00	1.00	1.00	1.00	0.14	–	-0.01
PLS	0.53	1.00	1.00	1.00	1.00	0.01	1.00	–
11 years	GLM	GLM-Net	SVM	RF	NN	k-NN	B-Trees	PLS
GLM	–	-0.01	-0.01	**-0.04**	-0.01	0.03	-0.03	-0.02
GLM-Net	1.00	–	-0.00	-0.03	0.00	**0.04**	-0.02	-0.01
SVM	0.91	1.00	–	-0.03	0.01	**0.04**	-0.02	-0.01
RF	0.01	0.35	0.23	–	0.03	**0.07**	0.01	0.02
NN	1.00	1.00	1.00	0.33	–	0.04	-0.03	-0.01
k-NN	0.21	0.04	0.03	0.00	0.17	–	**-0.06**	**-0.05**
B-Trees	0.09	1.00	1.00	1.00	1.00	0.01	–	0.01
PLS	1.00	1.00	1.00	0.11	1.00	0.01	1.00	–

Considering the performance of SVM, we notice that it is overall good, however it is slightly lower than the top pool of models. This could be explained by the fact that SVM is not designed to optimize AU-ROC as the prediction function is not calibrated by design in the SVM algorithm. In order to get a calibrated prediction output for SVM and other non-calibrated models we used the *softmax transformation* [22].

### Relative Importance

**Fig 4:**
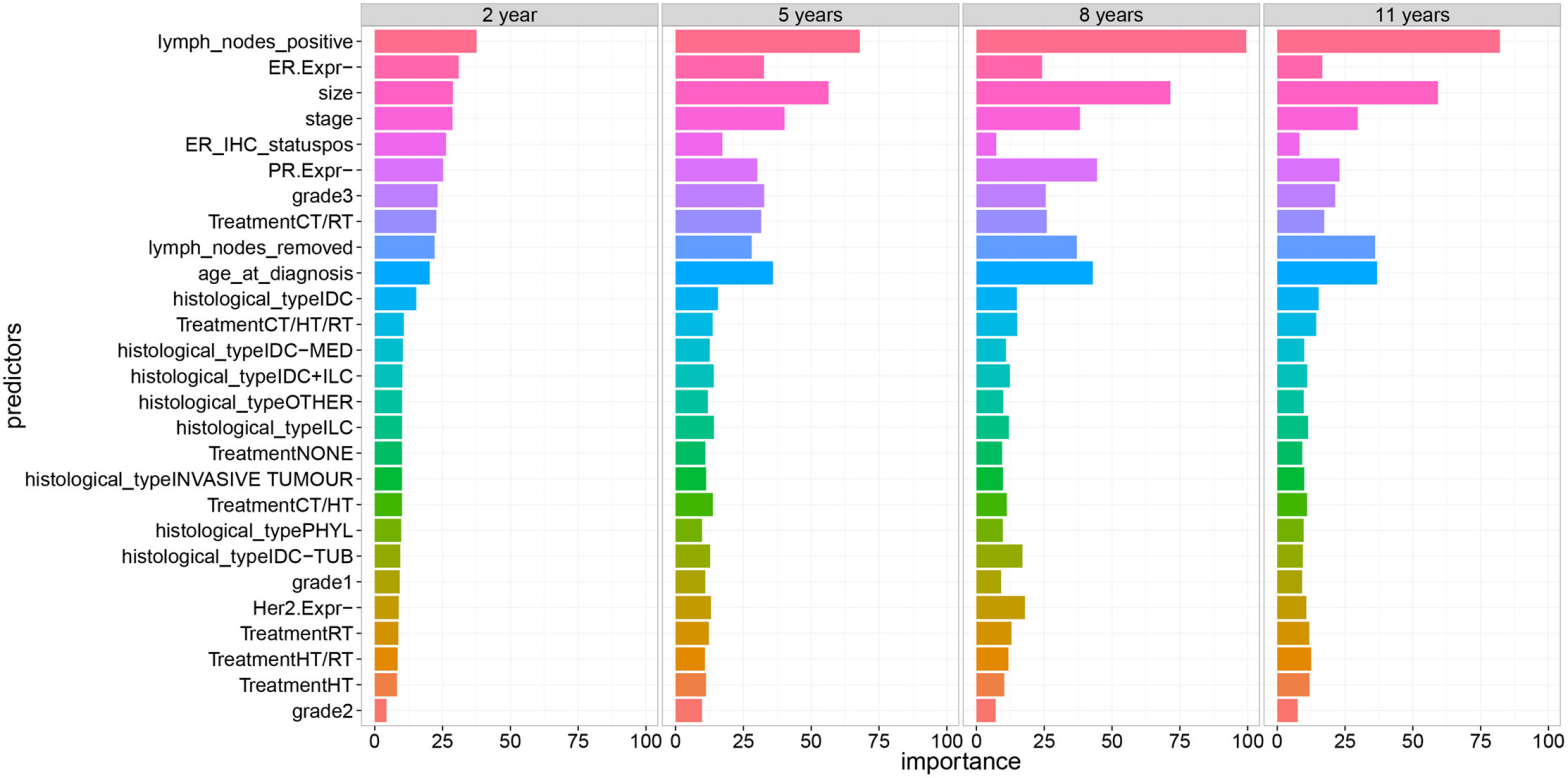
Normalized average relative variable importance for the different prognosis periods.

In addition to performance comparison, we analyzed the most important variables that contribute to the prediction models. For each model we quantified relative importance by giving a weight between 0 and 100 for each variable. We averaged the variable importance from the different models for the final analysis as shown in Fig 4 and Table 8. Models GLM, GLM-Net, Boosted Tree, Random Forests and PLS allow the derivation of variable importance during model training [16, 23, 24]. It is beyond the scope of this paper to provide the details of the derivation of variable importance for each model. For illustration purposes we describe briefly the derivation of variable importance for Random Forests model. In order to measure the relative importance of a certain variable, first Random Forests model is fitted on the training data and the out-of-bag error is estimated [25]. Then the values of this variable are perturbed in the training data and the out-of-bag error is estimated on the new perturbed dataset. The relative importance is therefore obtained as the average of the difference between the out-of-bag error before and after the perturbation over all trees. The score is finally normalized by the standard deviation of these differences [14]. The last procedure is repeated for the other variables to obtain the complete set of variable importance for Random Forests.

**Table 8:** Relative importance of the top 9 predictors for the different prognosis periods.

Predictors	2 years	5 years	8 years	11 years
lymph_nodes_positive	37.52	67.91	99.49	82.09
ER.Expr-	30.95	32.59	24.22	16.61
size	28.81	56.39	71.54	59.24
stage	28.65	40.19	38.17	29.68
ER_IHC_statuspos	26.28	17.24	7.30	8.23
PR.Expr-	25.18	30.15	44.47	22.97
grade3	23.15	32.66	25.54	21.38
TreatmentCT/RT	22.72	31.55	25.94	17.33
lymph_nodes_removed	22.06	28.06	37.02	36.08

For models for which it is not straightforward or not possible to extract variable importance, such as k-NN or SVM, we considered the use of AU-ROC as an alternative to evaluate the importance of each variable [24]. This can be obtained by considering single variable and then building an ROC using that variable as a predictor. Therefore variable importance is obtained for each predictor as the AU-ROC value. The obtained numbers are normalized across variables such that it ranges between 0 and 100 according to the following steps. The normalization is obtained by subtracting the min value of variable importance. The obtained values are then divided by the new max value. Therefore the variable with the largest variable importance will have a value of 100 corresponding to the maximum importance and lowest variable importance will correspond to 0. This normalization preserves the ranking of variables and allow the combination of results of variable importance from heterogeneous models. The final step in quantifying variable importance is to average the obtained values across models. Therefore predictors with an average variable importance close to 100 tend to be the most important variable for all models.

Fig 4 provides a plot of the average normalized relative importance for the different clinical variables and prognosis periods. Table 8 provides a numeric representation of Fig 4 corresponding to the same results. Variable importance values are sorted in a descending order for the prognosis period of 2 years. We can distinguish two sets of variables: The first set of variables has a strong influence on breast cancer prognosis while the second set has less impact on model predictability. The latter is mainly formed by variables related to the histological state, treatment types and the first two grades of the cancer. Concerning the first set of variables, the number of positive lymph nodes is the most important predictor for cancer prognosis across all prognosis periods with an average relative importance ranging from 37% to 99%. This predictor is highly important For mid and long-term prognosis and is the most important predictors for all models for the 8-year prognosis as it reached about 100%. Similarly, the number of positive lymph nodes, size, stage, PR.Expr and age at diagnosis variables have average relative importance of about 25%, but increase for mid and long-term prognosis. Tumor size is the second most important predictor for 5, 8 and 11 years with a relative variable importance of more than 50%. However its importance is around 25% for the short-term prognosis.

Variables such as Lymph node positive, Tumor size, Age and Stage are the most important predictors for T = 2 years. These predictors play also an important role for the other prognosis periods. However a predictor such as Treatment seems to have an important predictive factor for 5 years. Combined treatment Hormonal/Radio has slightly higher importance than other treatments. This result indicates that this treatment is important for improving mid-term breast cancer prognosis (5 years). However for long-term prognosis its corresponding relative importance drops to approximately 10%.

## Conclusion

We considered the problem of predicting breast cancer prognosis using clinical variables. We focused our analysis on the comparison of different classification techniques under the same resampling conditions. The best performing techniques have comparable results indicating that breast cancer prognosis can be made stable. The fitted models are used to extract relative importance of the different variables. The analysis of variable importance allows the quantification of the contribution of the clinical predictors in the prognosis models. We identified two sets of predictors: A first set that has a significant impact on prediction models. A second set that has a minor influence on prediction models. The most important predictors identified in the first set are: The number of positive lymph nodes, age at diagnosis, tumor size and cancer stage. These variables are important for the prediction across the different selected prognosis periods. This information can be used to improve the data collection quality of these parameters during clinical trials as they seem to be critical for the prediction. Concerning the effect of treatments on model prediction power, the analysis of relative variable importance reveals that Chemo/Radio therapy has an important mid-term prognosis effect. The remaining treatment types have limited effect on prognosis.

These results will go through a clinical validation step by sharing the results with clinicians and planning additional complementary analyses. Supplementary variables will be included to quantify and compare the importance of genomic versus clinical predictors. Since the number of genomic variables are usually much large than clinical variables, appropriate ways of combining both sets should be investigated. In order to further confirm the results of this paper we plan to benchmark the results with other breast cancer prognosis datasets. As a next step for model comparison, we will investigate different approaches to combine the models such as bagging, boosting and other ensemble techniques. We expect that the combined model should show better performance and higher robustness.
